# Improvement of the UV Barrier and Antibacterial Properties of Crosslinked Pectin/Zinc Oxide Bionanocomposite Films

**DOI:** 10.3390/polym13152403

**Published:** 2021-07-22

**Authors:** Karina Dyasti Hari, Coralia V. Garcia, Gye-Hwa Shin, Jun-Tae Kim

**Affiliations:** 1 Department of Food Science and Technology, Keimyung University, Daegu 42601, Korea; karina.dyastihari@gmail.com (K.D.H.); cvalentinag@yandex.com (C.V.G.); 2 Department of Food and Nutrition, Kunsan National University, Gunsan 54150, Korea; winnie19@kunsan.ac.kr

**Keywords:** antibacterial, bionanocomposite, pectin, zinc oxide, crosslinking

## Abstract

Pectin-based antibacterial bionanocomposite films were prepared by crosslinking with calcium chloride (CaCl_2_) and mixing with zinc oxide nanoparticles (ZnO-NPs) at various concentrations (0.5%, 1%, and 1.5% *w/w*, based on pectin). Crosslinking with 1% CaCl_2_ significantly (*p* < 0.05) improved the tensile strength of the pectin films, although their elongation at break was decreased. The UV-light barrier property of the pectin/ZnO bionanocomposite films was significantly (*p* < 0.05) improved with increasing ZnO-NP concentrations. In addition, the bionanocomposite films incorporating 1.5% ZnO-NPs showed excellent antibacterial effects against both *Escherichia coli* and *Staphylococcus aureus*, inhibiting over 99% of the bacteria. Therefore, the developed crosslinked pectin/ZnO bionanocomposite films show great potential as active packaging materials with excellent UV-blocking and antibacterial properties.

## 1. Introduction

In recent years, antimicrobial packaging has attracted a great deal of attention from the food industry because of the increase in the consumer demand for chemical preservative-free products [1]. Moreover, there is growing interest in biodegradable packaging as an eco-friendly replacement for petroleum-based polymers. As abundant, renewable, environmentally friendly, and sustainable materials, biopolymers have been used to fabricate biodegradable plastics and edible films [2,3]. Among biopolymers, polysaccharides are attractive materials because of their good film-forming properties, sustainability, and abundance [4,5]. 

Pectin is the major structural component of the plant cell wall and is one of the largest constituents in citrus by-products. This polysaccharide is generally recognized as safe (GRAS) by the United States Food and Drug Administration (FDA), and is used in food processing, mostly as a gelling, stabilizing, or thickening agent in products such as jams, yogurt, fruit drinks, and ice cream [6]. Pectin is mainly composed of galacturonic acid units. Depending on its degree of esterification (DE), pectin can be classified as high methoxyl pectin (DE > 50%) and low methoxyl pectin (DE < 50%), with both being able to be used for film formation [7]. Some of the advantages of pectin edible films are biocompatibility, biodegradability, and non-toxicity [8]. Nevertheless, neat pectin films do not show satisfactory functionality because of their high hydrophilicity, poor mechanical and barrier properties, and low water resistance [9,10]. Hence, various strategies have been tested for improving the properties of pectin-based films, including crosslinking, blending with other polymers, and incorporating nanoparticles and essential oil nanoemulsions [7,10]. Such films can also be engineered to exhibit functionalities. For instance, pectin films incorporating silica nanoparticles extended the shelf life of strawberries, and pectin/corn flour/beetroot powder films extended the shelf life of tomatoes [11,12]. Pectin films incorporating lime antioxidants hindered soybean oil oxidation [13]. Films made of a combination of pectin and hydrocolloids from chia seeds showed antioxidant properties, and electrospun pectin films were used in multilayer structures with aroma barrier properties [14,15]. Moreover, pectin films incorporating ZnO nanoparticles (ZnO-NPs) were reported to show enhanced strength and water barrier properties as well as antimicrobial effects [9].

Compared with neat pectin films, crosslinked pectin films exhibit increased tensile strength and water resistance [16]. The crosslinking mechanism of pectin with divalent metal ions such as Ca^2+^ and Zn^2+^ is explained by the “egg-box” model, in which the crosslinks are formed by divalent ions occupying electronegative cavities in the ribbon structure of carboxylic groups [17]. Hence, the functional and mechanical properties of pectin films could be improved by incorporating both calcium ions and ZnO-NPs. 

ZnO-NPs exhibit advantages such as non-toxicity, availability, low cost, high ultraviolet absorption capacity, and strong antimicrobial activity [18,19]. Compared to other natural antibacterial materials such as nisin, essential oils, grapefruit seed extract (GFSE), etc., ZnO-NPs have high thermal stability and do not lose their antibacterial activity during the processing of most food packaging films such as polyethylene (PE), polypropylene (PP), polyethylene terephthalate (PET), etc. It is thought that the antimicrobial activity of ZnO-NPs is dependent on the release of Zn^+2^ ions and the formation of reactive oxygen species (ROS), which damage the integrity of the microbial cells [20]. ZnO is recognized as a GRAS substance by the FDA, although no differentiation is made between the bulk and nano form. Moreover, ZnO-NPs are approved for certain food contact applications in the European Union [21].

Therefore, here, we aimed to optimize the development of crosslinked pectin-based films incorporating ZnO-NPs. The effects of calcium chloride and ZnO-NP concentration on the mechanical, physical, optical, UV barrier, and antibacterial ability of the bionanocomposite films were evaluated. In particular, the goal of this study was to produce bionanocomposite films with enhanced UV-blocking and antibacterial properties.

## 2. Materials and Methods

### 2.1. Materials

Pectin powder from citrus was purchased from Daejung Chemicals & Metals Co., Ltd. (Siheung, Korea). Glycerol was obtained from Duksan Pure Chemicals Co., Ltd. (Ansan, Korea). Calcium chloride (CaCl_2_) and ZnO nanopowder were purchased from Sigma Aldrich Chemicals (St. Louis, MO, USA). Nutrient broth (NB) was purchased from DB Difco^TM^ (Sparks, MD, USA). *Escherichia coli* ATCC 8739 and *Staphylococcus aureus* ATCC 6538 P were procured from the American Type Culture Collection (ATCC).

### 2.2. Film Preparation

#### 2.2.1. Crosslinked Pectin Films

To prepare crosslinked pectin films (CPF), 4 g of pectin was dissolved in 170 mL of distilled water. As a plasticizer, glycerol was added at 20% (*w/w*, based on pectin) to prevent brittleness. Then, a CaCl_2_ solution (30 mL; 0.5%, 1%, 2%, and 5% *w*/*v*) was added to the pectin solution and mixed using a magnetic stirrer at 70 °C for 20 min. The film-forming solution was cooled down at room temperature for 30 min before casting it onto a Teflon-coated glass plate (13.5 cm × 25 cm) and drying at 30 °C in a convection oven for 48 h. The fully dried pectin films were conditioned at 25 °C and 50% relative humidity (RH) for 48 h before testing their mechanical properties.

#### 2.2.2. Crosslinked Pectin/ZnO Bionanocomposite Films

For the preparation of crosslinked pectin/ZnO bionanocomposite films (CPZBF), a ZnO-NP dispersion was prepared by adding ZnO nanopowder (0.5%, 1%, and 1.5%, *w/w*, based on pectin) to 170 mL of distilled water containing 20% (*w/w*) glycerol, followed by ultrasonication at 40% amplitude for 10 min. 4 g of pectin was dissolved in the homogeneous dispersion of ZnO-NPs and stirred at room temperature for 3 h. Then, 30 mL of a CaCl_2_ solution (1% *w/w*, based on pectin) was added and mixed using a magnetic stirrer at 70 °C for 20 min. The film-forming solution was cooled down at room temperature for 30 min before casting it onto the Teflon-coated glass plate (13.5 cm × 25 cm) and dried as described in Section 2.2.1.

### 2.3. Characterization of Bionanocomposite Films

#### 2.3.1. Optical Properties

The color of the CPZBF was determined using a Chroma Meter (CR-400, Minolta, Tokyo, Japan). A white color standard plate (L* = 96.76, a* = 0.05, b* = 1.94) was used as a background. The total color difference (∆*E*) was calculated using Equation (1)

(1) $$\Delta E=\sqrt{{\left(\Delta L\right)}^{2}+{\left(\Delta a\right)}^{2}+{\left(\Delta b\right)}^{2}}$$

where Δ*L*, Δ*a*, and Δ*b* represent the differences in lightness (*L*), redness (*a*), and yellowness (*b*) values of the control (crosslinked pectin film) and CPZBF samples. At least five points were measured for the color parameters for each film, and the average values were used.

The UV absorbance of the CPZBF was measured using a UV–visible spectrophotometer (UV-2600, Shimadzu, Kyoto, Japan) at wavelengths of 200–550 nm.

#### 2.3.2. Mechanical Properties

The bionanocomposite films were cut into 10 mm × 10 mm strips and conditioned at 25 °C and 50% RH for 48 h before their mechanical test. Film thickness was measured in five random locations in each sample using a micro-caliper (MDC-25MJ, Mitutoyo Co., Kanagawa, Japan), and the average was calculated. Tensile strength, elongation at break, and Young’s modulus of the films were tested using a Universal Testing Machine (UTM; Zwick 2010TN, Zwick GmbH & Co. KG, Ulm, Germany) according to the standard method of ASTM D882-12 [22].

#### 2.3.3. Moisture Content

To determine the water content, the crosslinked pectin films were cut into 2 cm × 2 cm squares and weighed (*W*_1_). The water content of the films was determined by drying the film samples in a convection oven at 105 °C for 24 h, and weighing again (*W*_2_) after cooling them to room temperature. The moisture content was calculated using Equation (2):

(2) $$\mathrm{Moisture}\;\mathrm{content}\;(\%)=\frac{W_{1}-W_{2}}{W_{1}}\times 100$$

#### 2.3.4. Water Solubility

Water solubility was determined using a previously published method with slight modifications [23]. Film samples (2 cm × 2 cm) were dried at 105 °C in a convection oven for 24 h and weighed (*W_i_*). Then, the film samples were put into flasks (50 mL) containing 20 mL distilled water with constant stirring at 25 °C for 6 h. The liquid was filtered and the remaining film mass was dried at 105 °C for 24 h until it showed a constant weight (*W_f_*). Film solubility was calculated using Equation (3)

(3) $$\mathrm{Film}\;\mathrm{solubility}\;(\%)=\frac{W_{i}-W_{f}}{W_{i}}\times 100$$

#### 2.3.5. Water Vapor Permeability (WVP)

The water vapor permeability of the films was determined using a modified cup method [24]. Film samples were cut into 6.7 cm × 6.7 cm squares and covered the cups containing distilled water. Samples were stored in a chamber at 25 °C and 25% RH for 12 h. Changes in the weight of the film samples were recorded every 2 h. The water vapor permeability was calculated using Equation (4)

(4) $$\mathrm{WVP}=\frac{\mathrm{WVTR}\times \delta }{p_{1}-p_{2}}\times 100$$

where WVTR is the water vapor transmission rate, δ is the film thickness, and p_1_ and p_2_ are the partial pressures of water vapor inside and outside the cup, respectively.

#### 2.3.6. FTIR Analysis

The Fourier-transform infrared (FTIR) spectra of the films were recorded using attenuated total reflection Fourier transmission infrared (ATR-FTIR) equipment (Nicolet Is5, Thermo Fisher Scientific, Waltham, MA, USA). The film samples were cut into rectangular strips (4 cm × 4 cm) and were directly put onto the ATR cell. The spectra were recorded as 64 scans at a 4 cm^−1^ resolution ranging from 600 to 4000 cm^−1^ wavenumber.

#### 2.3.7. Differential Scanning Calorimetry (DCS)

Thermal properties of the films were evaluated using a DSC 25 (TA Instruments, New Castle, DE, USA) as previously reported, with modifications [25]. Roughly 5–10 mg of each film sample was put in an aluminum pan and heated from 0 to 300 °C at a constant heating rate of 10 °C/min under a nitrogen flow of 50 mL/min. DSC thermograms were recorded.

#### 2.3.8. Antibacterial Activity

The antibacterial activity of the bionanocomposite films was examined against *E. coli* (Gram-negative) and *S. aureus* (Gram-positive). The antimicrobial test was performed by the plate counting method with slight modifications [18]. *S. aureus* and *E. coli* were individually grown on NB at 37 °C. The sample films were cut into rectangular shapes (1 cm × 6 cm) and placed in test tubes. Diluted broth (10 mL, around 10^5^ CFU/mL) was poured into a test tube containing a film sample, and incubated at 37 °C for 24 h. Diluted broth alone was used as the control. After incubation, the cell viability of each bacterium was calculated by counting colonies on the plates, and reporting the values as CFU/mL. The antibacterial rate was calculated using Equation (5):

(5) $$\mathrm{Antibacterial}\;\mathrm{rate}\;(\%)=\frac{\left(\mathrm{Nc}-\mathrm{Ns}\right)}{\mathrm{Nc}}\times 100$$

where Nc is the number of viable bacteria on the control film (pure LDPE film) and Ns is the number of viable bacteria on the bionanocomposite films after 24 h of incubation. The antibacterial activity was calculated as the difference in logarithmic values of viable bacteria between the control film and bionanocomposite films as described in the JIS Z2801:2010 regulation [26].

### 2.4. Statistical Analysis

Experiments were duplicated at least three times, and data are expressed as mean ± standard deviation (SD). Statistical significance was determined by analysis of variance (ANOVA) and Duncan’s multiple range test. The level of significance was set at *p* < 0.05 using SPSS version 16 (SPSS Inc., Chicago, IL, USA).

## 3. Results and Discussion

### 3.1. Characterization of Crosslinked Pectin Films

#### 3.1.1. Mechanical Properties

Table 1 shows the effects of CaCl_2_ concentration on the thickness, tensile strength (TS), Young’s modulus (YM), elongation at break (*E*), moisture content, and water solubility of pure pectin (control) and crosslinked pectin films. Adding CaCl_2_ increased the thickness of the films as compared to the control film, with the films crosslinked with 1% CaCl_2_ having a thickness of 101.5 ± 11.9 μm and the control films of 85.1 ± 8.1 μm. The TS of pectin film crosslinked with 1% CaCl_2_ (22.7 ± 1.4 MPa) was significantly (*p* < 0.05) higher than that of the control (14.3 ± 1.4 MPa), although further increases in CaCl_2_ resulted in a slight but not significant (*p* > 0.05) decrease in TS. On the other hand, crosslinking caused a significant (*p* < 0.05) decrease in the *E* of the films, with the control and the film crosslinked with 1% CaCl_2_ having *E* values of 8.3% and 6.1%, respectively. Nevertheless, *E* was not significantly (*p* > 0.05) changed with further increases in the CaCl_2_ content.

The increase in TS by the addition of CaCl_2_ is due to the crosslinking between the carboxyl acid (COO^–^) of pectin and calcium ions (Ca^2+^), described in the “egg-box” model [27,28]. The multivalent cations penetrate the film matrix and enhance the formation of bonds between the polymer chains, resulting in an increase in TS but also less flexibility and a more rigid polymer structure. Based on the results obtained, the film crosslinked with 1% CaCl_2_ was used for further experiments.

#### 3.1.2. Moisture Content and Water Solubility

Crosslinking with CaCl_2_ decreased both the moisture content and water solubility of the pectin films (Table 1). The pure pectin film (control) had 19.6% ± 1.9% moisture, which significantly (*p* < 0.05) decreased to 14.2% ± 1.2% after the addition of 1% CaCl_2_. Nevertheless, further increases in CaCl_2_ did not significantly (*p* > 0.05) affect the moisture content. Water solubility showed the same trend, decreasing significantly (*p* < 0.05) from 51.3% ± 3.4% to 31.8% ± 2.8% after crosslinking with 1% CaCl_2_. CaCl_2_-crosslinked pectin resulted in decreased moisture content and water solubility, indicating that the Ca^2+^ ions promoted greater cohesion of the intermolecular bonds, leaving less available spaces for accommodating water molecules [29]. Crosslinking agents can thus help to overcome the poor moisture resistance of polysaccharide films by decreasing their hydrophilic properties through the formation of bonds between the polysaccharide molecules, reducing water absorption [30].

#### 3.1.3. FTIR Analysis

**Figure 1:**
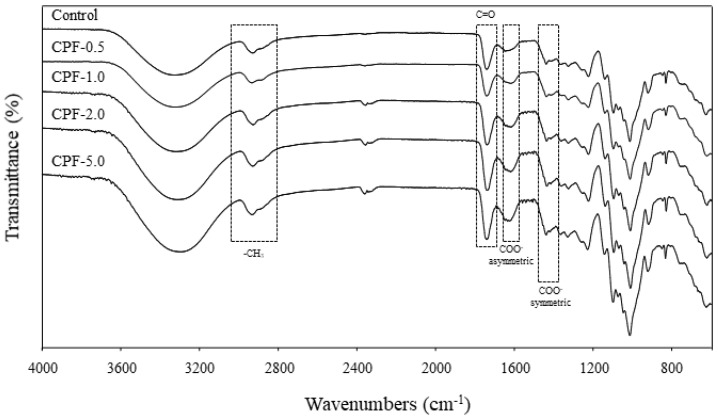
FTIR spectra of pure pectin film (control) and crosslinked pectin films (CPF) with 0.5, 1, 2, and 5% CaCl_2_.

The FTIR spectra of neat and crosslinked pectin films are shown in Figure 1. The main functional groups were identified as carbomethoxy (C=O, ~1740 cm^−1^), methyl (–CH_3_, ~2938 cm^−1^), and carboxylate (COO– asymmetric and symmetric stretching vibration at ~1630 cm^−1^ and ~1437 cm^−1^) [31]. The intensity of the C=O, –CH_3_, and COO–became higher with calcium concentrations, indicating that Ca^2+^ interacted with the C=O group of pectin [32]. In addition, the increase in intensity may also be due to the electrostatic interactions between Ca^2+^ and COO– according to the “egg-box” model, and demonstrate that crosslinking occurred effectively [27,33].

### 3.2. Characterization of Crosslinked Pectin/ZnO Bionanocomposite Films

#### 3.2.1. Surface Color and Optical Properties

**Figure 2:**
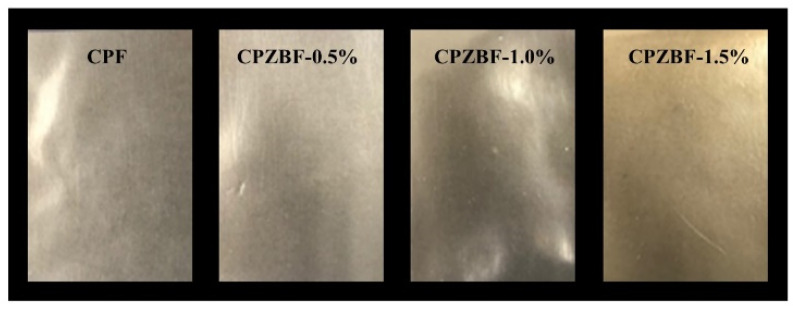
Photographs of the crosslinked pectin film (CPF) and the crosslinked pectin/ZnO bionanocomposite films (CPZBF) containing 0.5~1.5% ZnO-NPs.

Figure 2 shows the photographs of the crosslinked pectin film (CPF) and the crosslinked pectin/ZnO bionanocomposite films (CPZBF) containing 0.5~1.5% ZnO-NPs. The appearance of the composite films changed to a more yellow color with increasing ZnO-NP concentrations. The chromaticity of the bionanocomposite films is shown in Table 2. The control film (crosslinked pectin films without ZnO-NPs) was more transparent than the bionanocomposite films combined with ZnO-NPs. As expected, the total color difference (*ΔE*) in the bionanocomposite films increased with the addition of ZnO-NPs. As the content of ZnO increased, the *L* and *a* value decreased and the *b* value increased significantly (*p* < 0.05), corresponding to a decrease in transparency and increase in yellowness. Control films exhibited *L*, *a*, and *b* values of 93.3 ± 0.6, −0.23 ± 0.04, and 6.31 ± 0.67, respectively. By contrast, the *L*, *a*, and *b* values of the bionanocomposite film with 1.5% ZnO-NPs were 91.4 ± 0.2, −0.34 ± 0.03, and 8.52 ± 0.22, respectively. These changes were reflected in the more intense dark green coloration of the bionanocomposite films as the ZnO-NP concentration increased. The increase in opacity resulting from the incorporation of ZnO-NPs has been reported by other researchers [9,34].

**Figure 3:**
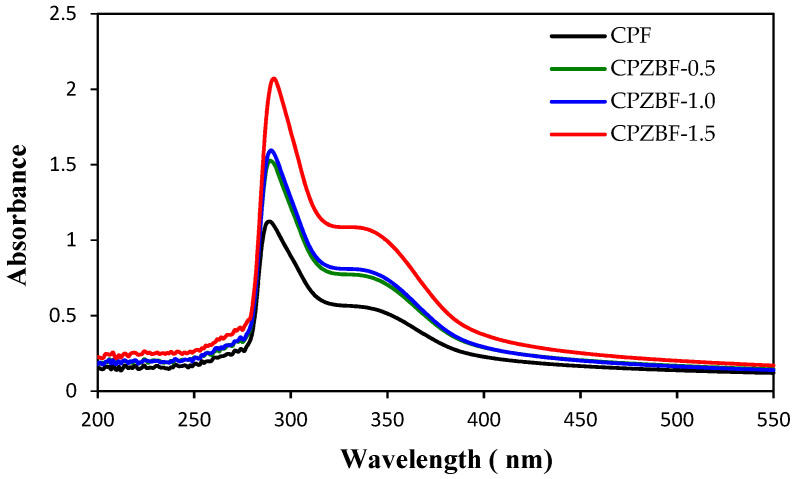
UV–visible spectra of crosslinked pectin film (CPF) and crosslinked pectin/ZnO bionanocomposite films (CPZBF) with 0.5, 1, and 1.5% ZnO nanoparticles.

The UV barrier properties of the bionanocomposite films were evaluated by measuring their absorbance in the wavelength range of 200–550 nm (Figure 3). The absorbance of the bionanocomposite films increased after the incorporation of ZnO-NPs. Furthermore, two absorbance peaks were visible, including a sharp peak in the 280–300 nm region, which is characteristic of pectin [35], and a broad peak at approximately 360 nm, corresponding to ZnO-NPs [12]. The increase in UV absorbance as a result of ZnO incorporation agrees with previous reports [18,34,36,37]. The results obtained indicate that incorporating ZnO-NPs into pectin improves the UV barrier properties of the resulting bionanocomposite films. Therefore, the developed bionanocomposite films could be used as UV-screening food packaging materials.

#### 3.2.2. Mechanical Properties

Table 3 shows the effects of the ZnO-NP concentration on the thickness, YM, TS, and *E* of the pectin/ZnO bionanocomposite films. Increasing the ZnO-NP concentration did not have significant (*p* > 0.05) effects on the bionanocomposite film thickness. However, the TS was significantly (*p* < 0.05) increased from 22.7 ± 1.4 MPa for the control to 24.6 ± 1.8 MPa for the bionanocomposite film with 1.5% ZnO-NPs. The Young’s modulus also increased from 857.3 ± 56.5 MPa for the control to 899.4 ± 65.8 MPa for the nanocomposite with 1% ZnO-NPs. On the other hand, *E* tended to decrease as the ZnO-NP content increased, from 6.1% ± 1.0% for the control to 4.9% ± 0.5% for the sample with 1.5% ZnO-NPs. The bionanocomposite films developed here were stronger than pea starch/ZnO nanocomposites (TS of 10.8 MPa) [37], although weaker than pectin/alginate/ZnO nanocomposites (TS of 41.6 MPa) [34] and pectin/ZnO nanocomposites incorporating 5% ZnO-NPs (TS of 45.4 MPa) [9].

The mechanical properties of bionanocomposite films can be influenced by several factors, including the dispersion of the nanofillers and nanofiller–matrix interactions [37]. Some studies agree with the observed increase in TS as a result of the incorporation of ZnO-NPs [33,36]; however, the opposite effect was reported for nanocomposites based on agar, carrageenan, carboxymethyl cellulose, and gelatin [18,36].

The increase in TS observed here suggests that Zn^2+^ binds to the hydroxyl (OH^–^) and carboxylate (COO^–^) groups of pectin chains in a similar way as that described in the “egg-box” model, although Ca^2+^ is reported to interact only with carboxylate groups [38]. The effectiveness of the reinforcement material and its dispersion in the polymer matrix are crucial for effective stress transfer at the matrix–nanofiller interface, and ultimately increase the TS of polymeric biocomposite materials, although the trade-off is a decrease in elasticity [39,40]. The TS of the bionanocomposite films developed is comparable to that of polypropylene and ethylene-polypropylene (26 MPa), although their *E* is substantially lower than that of most other polymers used in packaging except polystyrene (1.6%) [41]. Thus, further improvements in the mechanical properties of the bionanocomposite films are necessary.

#### 3.2.3. Water Vapor Permeability (WVP)

The WVP of the bionanocomposite films is shown in Table 3. No significant (*p* > 0.05) changes in the WVP of the films were observed, suggesting that the incorporation of ZnO-NPs did not affect the water affinity of the pectin-based bionanocomposite films. This result may be due to the high hydrophilicity of pectin, the low concentration of ZnO-NPs incorporated into the films, and the high concentration of glycerol used to prepare them, as it has been reported that glycerol may increase the spacing between polymer chains, enabling the passage of water vapor [29]. The WVP obtained here is higher than that of synthetic plastics as well as pure hydroxypropyl methylcellulose films [24], which are known for their hydrophilicity, and thus indicates that decreasing the WVP of the pectin/ZnO bionanocomposite films remains a challenge.

#### 3.2.4. DSC Analysis

**Figure 4:**
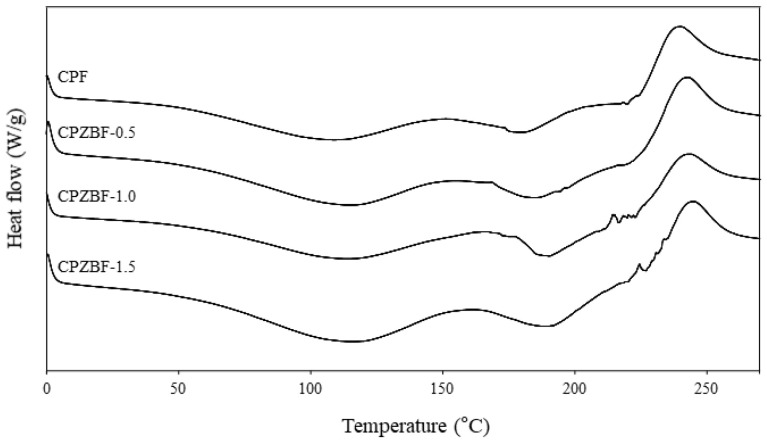
DSC thermograms of crosslinked pectin film (CPF) and crosslinked pectin/ZnO bionanocomposite films (CPZBF).

The DSC thermograms of the crosslinked pectin film and pectin/ZnO bionanocomposite films are shown in Figure 4. Only small changes were observed in the thermograms, suggesting that ZnO incorporation only slightly improved the thermal resistance of the films. This may be explained by the low concentration of ZnO incorporated [42]. The thermograms of the crosslinked pectin film (CPF) showed two endotherms, one at around 110 °C and the other at around 180 °C. These temperatures increased to 115 and 190 °C, respectively, for the bionanocomposite film with 1.5% ZnO (CPZBF-1.5). The first endotherm represents the glass transition temperature (T_g_) of the film and is associated with water loss, whereas the second one represents the melting temperature (T_m_) of the film [43,44]. In addition, an exothermic peak was observed at around 245 °C for the bionanocomposite film with 1.5% ZnO (240 °C for the crosslinked pectin film). This peak represents the thermal depolymerization of pectin [45] and, as the results show, the addition of ZnO hindered the degradation of the polymer, albeit slightly.

#### 3.2.5. Antibacterial Activity

**Figure 5:**
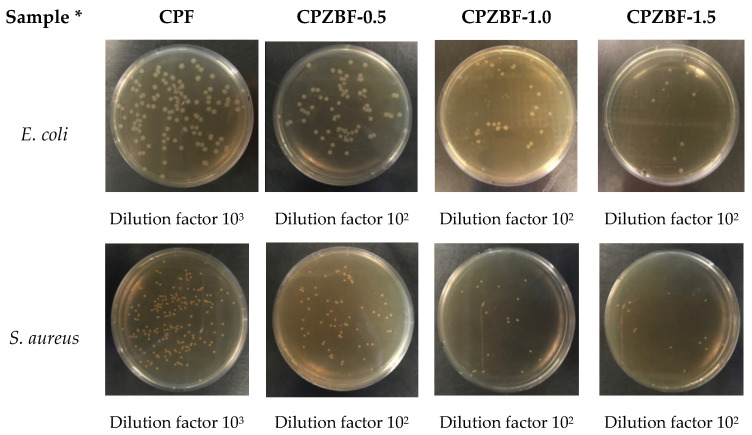
Colony forming units (CFU) of *E. coli* and *S. aureus* after exposure to crosslinked pectin film (CPF) and crosslinked pectin/ZnO bionanocomposite films (CPZBF). * Concentrations represent % ZnO (*w/w*).

The antibacterial activity of the bionanocomposite films against *E. coli* and *S. aureus* is shown in Table 4 and Figure 5. The bionanocomposite films showed antibacterial activity against both *E. coli* and *S. aureus*, although their effectiveness was slightly greater against the latter. Other studies have also reported the stronger antimicrobial effects of ZnO-NPs against Gram-positive bacteria [18,36]. Moreover, antifungal effects of ZnO-NPs were also reported [9]. The antibacterial activity of the bionanocomposite films increased significantly (*p* < 0.05) with an increase in the concentration of ZnO-NPs, with the bionanocomposite film containing 1.5% ZnO showing the greatest antibacterial rate (>99%) against both strains. At this concentration, the antibacterial activity was 2.04 ± 0.04 and 2.11 ± 0.05 for *E. coli* and *S. aureus*, respectively, indicating that the films were effective according to the Z2801:2010 regulation, which requires a minimum antibacterial activity of 2.0 [26].

The antibacterial properties of ZnO-NPs have been reported by other researchers [18,36,42,46]. The antibacterial activity of ZnO-NPs appears to be due to the release of Zn^2+^ ions, which penetrate the cell wall of bacteria, killing them. Because Gram-positive bacteria have a thick cell wall to which ZnO-NPs can bind, they are more susceptible to these antibacterial effects. ZnO-NPs can also generate reactive oxygen species (ROS) such as hydrogen peroxide (H_2_O_2_), which damage the cell membrane of bacteria [47]. It is assumed that both the production of ROS and the deposition of Zn^2+^ ions within the cytoplasm lead to either the inhibition or killing of bacterial cells [20,48]. Thus, the films developed in this study could be applied as wrapping to hinder bacteria and extend the shelf life of fresh produce, as reported in the literature for similar films [7]. Considering the lower mechanical and water barrier properties of pectin films compared to polymers commonly used in packaging, another strategy to be considered would be to incorporate these films into multilayer structures, aiming to provide specific functions, namely antimicrobial effects and UV-barrier properties, to the packaging [15,20].

## 4. Conclusions

Crosslinked pectin films showed greater tensile strength than did pure pectin films, although their elongation at break was lower. FTIR analysis revealed the interaction between pectin and CaCl_2_ and confirmed the formation of crosslinking. Adding ZnO-NPs to the crosslinked pectin films significantly improved their UV barrier and mechanical properties, resulting in stronger bionanocomposite films able to block UV irradiation, although the trade-off of this outcome was darkening and loss of flexibility. In addition, the crosslinked pectin/ZnO bionanocomposite films exhibited strong antibacterial activity against food-borne pathogenic bacteria such as *E. coli* and *S. aureus*. Therefore, the developed bionanocomposite films show potential to be used as active food packaging with antibacterial and UV-light barrier properties.

## Figures and Tables

**Table 1:** Effect of CaCl_2_ concentration on the mechanical properties of the crosslinked pectin films (CPF).

Sample *	Thickness (µm)	Young’s Modulus (MPa)	Tensile Strength (MPa)	Elongation at Break (%)	Moisture Content (%)	Water Solubility (%)
Control	85.1 ± 8.1 ^a^	535.8 ± 54.8 ^a^	14.3 ± 1.4 ^a^	8.3 ± 1.1 ^c^	19.6 ± 1.9 ^b^	51.3 ± 3.4 ^b^
CPF-0.5%	90.6 ± 10.3 ^a^	721.8 ± 34.5 ^b^	18.3 ± 1.4 ^b^	7.4 ± 0.8 ^b^	18.3 ± 0.7 ^b^	47.8 ± 1.0 ^b^
CPF-1.0%	101.5 ± 11.9 ^b^	857.3 ± 56.5 ^c^	22.7 ± 1.4 ^c^	6.1 ± 1.0 ^a^	14.2 ± 1.2 ^a^	31.8 ± 2.8 ^a^
CPF-2.0%	93.5 ± 17.9 ^a^	833.2 ± 121.6 ^c^	22.0 ± 2.5 ^c^	5.7 ± 0.7 ^a^	15.9 ± 0.7 ^a^	33.4 ± 0.8 ^a^
CPF-5.0%	88.1 ± 6.4 ^a^	763.3 ± 95.5 ^b^	21.9 ± 1.4 ^c^	5.6 ± 1.0 ^a^	16.0 ± 1.3 ^a^	32.6 ± 2.6 ^a^

* Concentrations represent % CaCl_2_ solution (*w*/*v*). Data represent the mean ± standard deviation. Different letters in the same column indicate significant differences at *p* < 0.05 by Duncan’s multiple range test.

**Table 2:** Effect of ZnO concentration on the surface color of the crosslinked pectin/ZnO bionanocomposite films (CPZBF).

Sample *	*L*	*a*	*b*	∆*E*
CPF	93.3 ± 0.6 ^c^	−0.23 ± 0.04 ^c^	6.31 ± 0.67 ^a^	-
CPZBF-0.5%	92.1 ± 0.6 ^b^	−0.29 ± 0.03 ^b^	8.07 ± 0.54 ^b^	2.18 ± 0.74 ^a^
CPZBF-1.0%	91.9 ± 0.5 ^b^	−0.36 ± 0.03 ^a^	8.16 ± 0.54 ^bc^	2.35 ± 0.65 ^a^
CPZBF-1.5%	91.4 ± 0.2 ^a^	−0.34 ± 0.03 ^a^	8.52 ± 0.22 ^c^	2.96 ± 0.25 ^b^

* Concentrations represent % ZnO (*w/w*). Data represent the mean ± standard deviation. Different letters in the same column indicate significant differences at *p* < 0.05 by Duncan’s multiple range test.

**Table 3:** Effect of ZnO concentration on the mechanical and water vapor barrier properties of the crosslinked pectin/ZnO bionanocomposite films (CPZBF).

Sample *	Thickness (µm)	Young’s Modulus (MPa)	Tensile Strength (MPa)	Elongation at Break (%)	WVP (mm·g/m^2^ kPa·h)
CPF	101.5 ± 11.9 ^a^	857.3 ± 56.5 ^ab^	22.7 ± 1.4 ^a^	6.05 ± 1.01 ^b^	3.71 ± 0.07 ^a^
CPZBF-0.5%	97.9 ± 13.9 ^a^	838.8 ± 70.4 ^a^	24.1 ± 2.2 ^ab^	5.92 ± 0.89 ^b^	3.45 ± 0.09 ^a^
CPZBF-1.0%	95.4 ± 15.6 ^a^	884.0 ± 48.4 ^b^	24.4 ± 2.1 ^b^	4.94 ± 0.65 ^a^	3.56 ± 0.08 ^ab^
CPZBF-1.5%	106.0 ± 13.2 ^a^	899.4 ± 65.8 ^b^	24.6 ± 1.8 ^b^	4.87 ± 0.49 ^a^	3.63 ± 0.14 ^ab^

* Concentrations represent % ZnO (*w/w*). Data represent the mean ± standard deviation. Different letters in the same column indicate significant differences at *p* < 0.05 by Duncan’s multiple range test.

**Table 4:** Antibacterial rate and activity of the crosslinked pectin/ZnO bionanocomposite films (CPZBF) against *E. coli* and *S. aureus*.

Sample *	*E. coli*		*S. aureus*	
	Antibacterial Rate (%)	Antibacterial Activity	Antibacterial Rate (%)	Antibacterial Activity
CPF	1.9 ± 0.2 ^a^	0.01 ± 0.01 ^a^	0.9 ± 0.5 ^a^	0.00 ± 0.01 ^a^
CPZBF-0.5	94.1 ± 0.4 ^b^	1.23 ± 0.03 ^b^	96.1 ± 0.3 ^b^	1.41 ± 0.03 ^b^
CPZBF-1.0	98.2 ± 0.2 ^c^	1.74 ± 0.05 ^c^	99.0 ± 0.1 ^c^	1.98 ± 0.05 ^c^
CPZBF-1.5	99.1 ± 0.1 ^c^	2.04 ± 0.04 ^d^	99.2 ± 0.1 ^c^	2.11 ± 0.05 ^d^

* Concentrations represent % ZnO (*w/w*). Data represent the mean ± standard deviation. Different letters in the same column indicate significant differences at *p* < 0.05 by Duncan’s multiple range test.

## Data Availability

All the data will be available to the readers.
